# Italian validation of the body odor disgust scale

**DOI:** 10.3389/fnbeh.2024.1389905

**Published:** 2024-06-21

**Authors:** Marco Tullio Liuzza, Marta Z. Zakrzewska, Jonas K. Olofsson

**Affiliations:** ^1^Department of Medical and Surgical Sciences, “Magna Graecia” University of Catanzaro, Catanzaro, Italy; ^2^Department of Clinical Neuroscience, Karolinska Institutet, Solna, Sweden; ^3^Department of Psychology, Stockholm University, Stockholm, Sweden

**Keywords:** behavioral immune system, body odor, gender difference, disgust, validation study

## Abstract

**Introduction:**

Disgust sensitivity to body odors plays a role in a set of psychological mechanisms supposedly evolved to avoid pathogens. To assess individual differences in body odor disgust, we previously developed the body odor disgust scale (BODS) and validated it in English. The BODS presents six scenarios where disgust could be evoked by smells coming from an internal source and an external source. The present study aimed to validate the BODS in the Italian population and to find further evidence for its structural, construct, and criterion validity.

**Methods:**

We used two large samples (*N* = 1,050, *F* = 527; and *N* = 402, *F* = 203, respectively) that were representative of the Italian population for sex and age.

**Results:**

Across these two studies, we confirmed the hypothesized bifactor structure, with all the items loading onto a general body odor disgust sensitivity factor, and on two specific factors related to the internal structure. In terms of construct validity, we found that the BODS converged with pathogen disgust sensitivity of the three-domain disgust scale (TDDS) but was distinct from a general propensity to experience negative emotions. The BODS showed criterion validity in predicting the behavioral intentions toward COVID-19 avoidance behavior, although it did not seem to be incrementally valid when compared to the TDDS pathogen subscale. We also established scalar measurement invariance of the BODS regarding gender and found that women display higher levels of BODS.

**Discussion:**

Results from the Italian version of the BODS indicate its structural, construct, nomological and criterion validity. Furthermore, our result on sex differences in disgust sensitivity are consistent with previous literature, and we discuss them in the broader context of cross-cultural and primate findings that points toward a possible evolutionary explanation of this difference.

## 1 Introduction

Disgust is a universal basic emotion characterized by a distinctive facial expression (Ekman, 1992) that has supposedly evolved to protect us from pathogenic substances (Rozin and Fallon, 1987) and pathogens (Oaten et al., 2009). The biological function of disgust is inferred from experimental evidence (e.g., Curtis et al., 2004) that pathogen-associated stimuli (e.g., a towel with a stain depicted in reddish yellow representing blood and bodily secretions) are perceived as more disgusting relative to the control stimulus (e.g., a towel with a blue stain). For this reason, the emotion of disgust has been placed at the core of the functioning of the so-called behavioral immune system (BIS, Schaller and Park, 2011), a set of psychological mechanisms evolved to detect, emotionally react, and avoid pathogen threats. Recent evidence indicates that people higher in pathogen disgust sensitivity are indeed less likely to be infected by viruses and bacteria (Cepon-Robins et al., 2021). An important aspect of disgust research is the validity of disgust-assessing instruments, their psychometric properties, and factorial structure. In the present study, we evaluate the body odor disgust scale (BODS) assessment in the Italian population.

Disgust and other negative emotions, such as fear, obey the principles outlined in error management theory (Haselton and Nettle, 2006); they lead people to follow a “better safe than sorry” strategy when the costs at stake (e.g., death) are overwhelmingly higher than the benefits. Disgust is thus likely to be activated by false positives. For instance, we are easily disgusted by food that has been taken before feeling sick, even if that food had nothing to do with the following state of disease (i.e., the “Garcia effect,” Garcia and Koelling, 1966). Moreover, it is harder to unlearn an association between a neutral stimulus and disgust than fear (see a recent meta-analysis by Mitchell et al., 2024). Similarly, disgust may be triggered by stimuli that are not directly associated with pathogen threats, such as obese people (Park et al., 2007), outgroup members from unfamiliar groups (Faulkner et al., 2004; Zakrzewska et al., 2019, 2020, 2023a; O’Shea et al., 2020; see Liuzza, 2020 for a review), or members of sexual minorities (Inbar et al., 2009, 2012; van Leeuwen et al., 2023).

People vary substantially in disgust sensitivity, the extent to which they emotionally react to disgusting stimuli (Tybur et al., 2018; Tybur, 2021). One of the first and widely used measures of disgust sensitivity was developed by Haidt et al. (1994). The disgust scale was created using a data-driven procedure that resulted in a 32-item instrument tapping into eight domains: food, sex, body products, body envelope violations, animals, hygiene, death, and sympathetic magic. However, the internal consistency of data was insufficient for this domain structure, leading to a revised 25-item scale (Olatunji et al., 2007) that tapped into three domains of disgust sensitivity: contamination-based, animal reminder, and core disgust. However, results in these domains are highly correlated, and the reliability of the contamination-based disgust domain is unsatisfactory. As an alternative to the DS-R, Tybur et al. (2009) developed the three-domain disgust scale (TDDS), a theory-driven instrument that is grounded in an evolutionary psychology framework. Indeed, Tybur and colleagues posited that apart from pathogen disgust, two other disgust domains evolved to solve distinct adaptive problems: sexual disgust, which serves the function of preventing suboptimal reproductive strategies, and moral disgust, which prevents interactions with an individual who may have a strong negative impact on ourselves or our communities (Tybur et al., 2009).

Although olfactory-induced disgust plays a marginal role in the most widely used scales of disgust sensitivity (see Liuzza et al., 2017a), the sense of smell is intimately related to this emotion (Liuzza, 2021). The emotional expression associated with disgust mimics the expulsion of putatively distasteful substances from the mouth (Darwin, 1872) and the wrinkling of the nose minimizes the air intake from the nose (Susskind et al., 2008). While taste, as noted by Rozin et al. (2009), primarily triggers disgust to prevent the ingestion of harmful substances, and olfaction, according to Stevenson (2010), offers an early detection system for microbial threats. A disgusting smell can alert us about dangers such as spoilage or toxic substances before they come into direct contact with our bodies. Together, these chemical senses form a comprehensive defense mechanism, highlighting the intricate ways by which humans avoid environmental threats to maintain health. Patients with olfactory impairment are more prone to consuming spoiled food, as noted by Temmel et al. (2002), due to their diminished ability to detect odors. Olfactory impairment also contributes to personal hygiene issues, stemming from an inability to self-monitor body odor. Disgust is primarily triggered by unpleasant smells (Alaoui-Ismaïli et al., 1997a,b; Bensafi et al., 2002; Croy et al., 2011). Odor-related disgust is particularly resilient to cognitive reinterpretation and harder to suppress than visual disgust (Adolph and Pause, 2012; Ferdenzi et al., 2013), highlighting the potent influence of olfaction in disgust.

Elicitors of disgust may vary from culture to culture (Herz, 2012). However, ethnographic surveys have found that some of these elicitors are universal—including odors associated with the body and bodily waste (Curtis and Biran, 2001). Body odors play an important role in regulating social interactions (Low, 2006) and in interpersonal communication (Boesveldt and Parma, 2021). Body odors are affected by diseases (Shirasu and Touhara, 2011), and humans react more negatively to the odors of individuals undergoing experimentally induced inflammatory responses (Olsson et al., 2014; Sarolidou et al., 2020; Gordon et al., 2023; Tognetti et al., 2023). In summary, the sense of smell likely plays a key role in the behaviors encompassed by the BIS framework. Given the aptness of body odors as a disease cue, disgust sensitivity to body odors should be particularly relevant for any evolved disease avoidance mechanism.

We developed an instrument specifically aimed at measuring individual differences in body odor disgust sensitivity (BODS, Liuzza et al., 2017a). The body odor disgust scale (BODS) consists of 12 items representing six distinct body odors: feces, upper body sweat, feet, urine, gas, and breath. Each type of odor is presented in scenarios reflecting both internal (experiencing one’s body odors, e.g., “You are alone at home and notice that your feet smell strongly”) and external (experiencing others’ body odors, e.g., “You are sitting next to a stranger and notice that their feet smell strongly”) source. Participants are asked to rate their disgust level for each scenario using a Likert-type scale that ranges from 1, indicating no disgust at all, to 5, signifying extreme disgust. This scale provides a nuanced tool for researchers to quantify disgust sensitivity toward body odors, allowing for the examination of how such sensitivities influence interpersonal relationships, personal hygiene practices, and potentially even mate selection and social dynamics.

The BODS was validated in English on US samples, and the results indicated good psychometric properties in terms of factor structure, measurement invariance conditional on gender, construct validity, and reliability (Liuzza et al., 2017a). In previous studies, we also provided criterion validity (Liuzza et al., 2017b; Zakrzewska et al., 2023b). In terms of nomological validity, the BODS also predicted relevant outcomes, such as explicit (Zakrzewska et al., 2019, 2023a) and implicit xenophobia (Zakrzewska and Liuzza et al., 2019), authoritarianism (Liuzza et al., 2018), and moral harshness (Liuzza et al., 2019). Importantly, in the study on the relationship between the BODS and xenophobia in a US sample, we also refined the measurement model of the BODS, as we found it better modeled by a bifactor model with a general BODS factor, and two specific factors, one for the internal, and one for the external source. Moreover, additional similarity between the pair of items from the same scenario (e.g., “You are alone at home and pass gas. It is silent but smells strongly” has some residual similarity with “You are sitting next to a stranger and they pass gas. It is silent but smells strongly”) was accounted for by modeling residual covariance.

In a recent cross-national study on nine different countries (Italy, Sweden, Canada, Chile, Hong Kong, Kenya, Nigeria, New Zealand, and the United Kingdom; Zakrzewska et al., 2023a), we found preliminary evidence for the validity of the BODS in these countries. However, in this study, we capitalize on previously collected datasets (one from the aforementioned study and one from an unpublished study) to validate the BODS more thoroughly in the Italian population. We aimed to (1) confirm the factor structure found earlier, test for (2) measurement invariance, (3) construct validity, and (4) criterion validity in an Italian sample. The studies were conducted according to the Declaration of Helsinki and were additionally approved by the National Ethics Review Authority in Sweden (Etikprövningsmyndigheten; 2018/1169–31/5 and 2020–04690).

## 2 Study 1

### 2.1 Materials and methods

#### 2.1.1 Participants

In Study 1, we analyzed the responses to the Italian BODS collected as a part of a larger published study (Zakrzewska et al., 2023a, the first wave of data collection). All participants completed the survey in Italian and provided their consent to their participation. Data were collected between 26/03/2020 and 01/04/2020 through the Qualtrics platform. We recruited participants between 18 and 70 years old, and the sample was representative of the Italian population for sex and age. Participants were paid 4 € each for their participation in the study. All participants provided consent to the participation. The median duration of the survey was 8 min and 10 s. In total, 1,050 participants declared to reside in Italy and completed the survey in Italian (*F* = 527).

#### 2.1.2 Measures

##### 2.1.2.1 Demographics

We collected demographic information from participants: whether they identify as female, male, or other; age; educational attainment; country of birth; and self-reported political orientation from 1 (*extremely left-wing*) to 7 (*extremely right-wing*).

##### 2.1.2.2 BODS

The BODS is a 12-item scale that measures disgust sensitivity to body odors (Liuzza et al., 2017a). Items refer to six types of body odors (feces, upper body sweat, feet, urine, gas, and breath) from either an internal (e.g., ‘You are alone at home and notice that your feet smell strongly’) and external (e.g., ‘You are sitting next to a stranger and notice that their feet smell strongly’) contexts. Participants rated the extent to which each scenario elicited disgust on a Likert-type response format from 1 (*not disgusting at all*) to 5 (*extremely disgusting*). The instrument was translated by the first author, who is a native Italian speaker who is fluent in English.

##### 2.1.2.3 Attention check

At the end of the survey, we included an attentional check, in which participants had first to read a longer text about free-time activities. The paragraph ended by instructing the participants to ignore the text, choose ‘Other’ as an answer, and type ‘I have read the instructions’ (or ‘OK’ in the second wave) in the text box that appeared. In total, 151 participants failed the attention check and were removed from the analyses. Such screening might also help limit the increasingly pervasive impact of artificial intelligence (AI) in survey responses (e.g., Webb and Tangney, 2022).

### 2.2 Data analysis

All the analyses were performed using R (R Core Team 2023), and RStudio (R Studio Team).

#### 2.2.1 Confirmatory factor analysis

**Figure 1:**
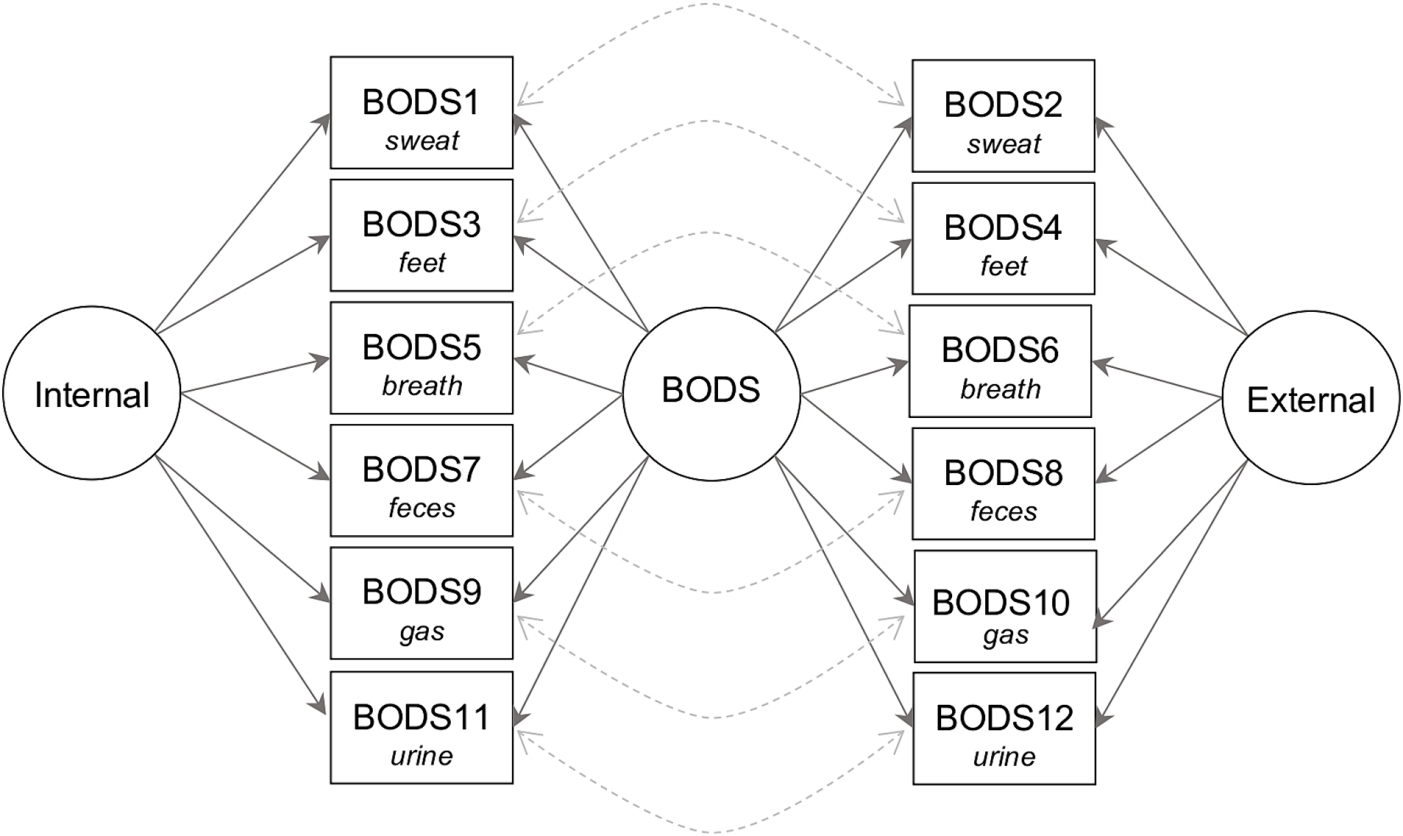
Path plot of the bifactor model of body odor disgust sensitivity (BODS). Circles represent latent variables: BODS—the overall body odor disgust sensitivity, Internal—disgust sensitivity toward body odors from internal sources (the internal subscale), and External—disgust sensitivity toward body odors from external sources (the external subscale). The squares represent observed variables (items on the scale). The solid arrows show that the item reflects latent variables, and the dashed arrows show how we modeled covariances between items pertaining to the same source.

We run a Confirmatory factor analysis (CFA) on the BODS using the *lavaan* package (Rosseel, 2012) to ensure that the scale is unidimensional. We followed Hu and Bentler (1999) for fit indices criteria: the comparative fit index (CFI) ≥ 0.9, Tucker–Lewis Index (TLI) ≥ 0.9, root mean square error of approximation (RMSEA) ≤ 0.08, and standardized root mean squared residual (SRMR) ≤ 0.10. In case of poor fit, we inspected the modification indices (MIs) and re-specified the model whenever the suggestions coming from the MIs were theoretically sound. For instance, the decision to model residual covariance was based on the fact that, after controlling for the variance explained by the latent factor, the residual covariance could be due to some semantic overlapping. The CFA was based on what we learned in previous studies (Liuzza et al., 2017a,b; Zakrzewska et al., 2019). In particular, we fitted a bifactor model with a general BODS factor, and two specific factors, one for the internal, and one for the external source. Moreover, since there was additional similarity between the pair of items to be accounted for (e.g., “You are alone at home and pass gas. It is silent but smells strongly” has some residual similarity with “You are sitting next to a stranger and they pass gas. It is silent but smells strongly”), we modeled residual covariance among pairs of items describing the same scenarios. Figure 1 illustrates the bifactor model. To decide whether to use a maximum-likelihood estimator (ML) or a robust maximum-likelihood estimator (MLR), we tested whether the data followed a multivariate normal distribution using the Mardia Test (Mardia, 1970) in the semTools package (Jorgensen et al., 2024). In the case of a significant departure from the multivariate normal distribution, we used MLR. We also estimated whether the scale reached acceptable reliability based on the omega total (𝜔*_t_* ≥ 0.6) and omega hierarchical (𝜔_ℎ_ ≥ 0.6) directly from the CFA model through the *compRelSEM* from the *semTools* package (Jorgensen et al., 2024).

#### 2.2.2 Measurement invariance for gender

We tested whether the BODS was invariant depending on gender. This would ensure that any difference between males and females in the BODS genuinely reflects differences in the latent variables. The data met the criteria for at least weak (metric) measurement invariance. Measurement invariance (MI) tests the assumption that the construct (latent variable), in this case, the BODS, is being measured in the same way across groups, in this case, gender. First, we aimed at assessing configural invariance, namely, the assumption that the measure holds the same structure across groups. Then, we aimed at testing metric (or weak) invariance, namely, that the indicators (items) have the same loadings onto the latent variable or, in other words, reflect the construct with the same strength. Third, we aimed at testing scalar invariance, namely, that the intercepts are equal across groups or, in other words, that when keeping the levels of the latent variable value at zero, the observed variable has the same values across groups. Whereas configural variance is an essential requirement for claiming that we are measuring the same constructs across groups, it is important to establish metric invariance if we want to compare the relationship between observed scores of the BODS and other variables across groups. In contrast, scalar invariance is crucial for having meaningful comparisons between groups. In practice, MI is tested by comparing nested CFA models when each of the constraints mentioned earlier is added to the model (see our analysis script on OSF for model definitions). MI may be considered rejected when the comparison between two models leads to a significant (*p* < 0.05) Δ𝜒2. However, since this method is prone to reject MI even in the presence of trivial non-invariance (Hays et al., 2005), in case of significance, we rejected MI when ΔCFI ≤ − 0.010, and ΔRMSEA ≥0.015 or ΔSRMR ≥0.030, when assessing metric invariance, and ΔCFI ≤ − 0.010, and ΔRMSEA ≥0.015 or ΔSRMR ≥0.010 when assessing scalar invariance (Chen, 2007). Provided sufficient invariance, we wanted to compare the BODS score for the two sexes using a t-test. Results are reported as mean difference with a 95% Confidence interval (CI).

### 2.3 Results

In the final sample (*N* = 899, *F* = 436) age spanned from 18 to 70 (mean = 43.08, SD = 12.77). The age distribution in this sample departed from the distribution of the Italian population (see Table 1), as retrieved from the National Institute of Statistics (ISTAT) 2023^1^. In particular, our sample under-represented people aged 56–70 years. In terms of educational attainment, the sample has a proportion of people with at least a bachelor’s degree (38.5%). Narrowing the analysis only to the participants aged 18–64 years, the proportion of the sample holding at least a bachelor’s degree is much higher (39.2%) than in the Italian population aged 16–64 years (20.1%, source: ISTAT).

**Table 1:** Demographic information about the sample in Study 1 and Study 2, and from the Italian population (source: ISTAT, and OCSE).

**Age group (%)**		**Study 1**	**Study 2**	**Italian population**
	18–24	7.6	10.1	8.6
	25–34	20.8	16.0	14.3
	35–44	26.0	19.8	18.9
	45–55	20.9	23.1	19.6
	55+	24.7	31.0	38.6
**Education level (%)**				
	Elementary	<0.1	-	6^a^
	Middle school	7.3	4.2	33
	High School	53.9	59.8	41
	BA/BSc	14.0	11.1	5^b^
	MA/MSc	20.1	22.3	14
	PhD	4.4	2.4	NA

Educational attainment from the Italian population refers to the age 25–64 years, whereas our sample ranged between 18 and 70. ^a^We collapsed Elementary (5%) and no qualification (1%) at all; ^b^We collapsed BA/BSc (4%) and non-academic post-high-school qualifications.

#### 2.3.1 Confirmatory factor analysis on the BODS

The Mardia test for multivariate normality showed that our data were not normally distributed both in terms of skewness (3062.53, *p* < 0.001) and kurtosis (65.83, *p* < 0.001). Therefore, we used a maximum likelihood robust (MLR) estimator. The CFA model showed a good fit (𝜒2 = 200.09, df = 36, CFI robust = 0.97, TLI robust = 0.94, SRMR = 0.04), except for RMSEA (RMSEA robust = 0.09), which was still acceptable. On top of the good global fit, the local fit looked acceptable as well, as all the items had loadings on the general factor ranging from 0 to 1. Table 2 shows item loadings onto the general factor, as well as the two specific factors (external and internal source). Regarding reliability, the scale showed excellent internal consistency (𝜔*_t_* = 0.92, 𝜔_ℎ_ = 0.76). Using recommendations from Reise et al. (2013), we find some support for the existence of an essentially unidimensional model: (𝜔_ℎ_ = 0.75 and explained common variance (ECV) = 0.65, even though the percent uncontaminated correlations (PUC) value is lower than the threshold suggested (PUC = 0.55).

**Table 2:** Item loadings for general factors (BODS, body odor disgust sensitivity) and the specific factors: external and internal BODS in Study 1.

**#**	**Item**	**Estimate (Std)** **95% CI**		
		**BODS**	**External**	**Internal**
3	You are alone at home and notice that your feet smell strongly.	0.87 [0.84, 0.9]	–	−0.02 [−0.21, 0.16]
5	You are alone at home and notice that your breath smells strongly.	0.81 [0.78, 0.84]	–	0.04 [−0.11, 0.19]
1	You are alone at home and notice that the t-shirt you are wearing smells strongly from your own sweat	0.8 [0.75, 0.85]	–	0.36 [0.22, 0.5]
11	While alone at home, you use the bathroom. Afterwards, you notice that the room smells strongly of your urine.	0.72 [0.65, 0.78]	–	0.31 [0.19, 0.43]
7	While alone at home, you use the bathroom. Afterwards, you notice that the room smells strongly of your feces.	0.7 [0.63, 0.77]	–	−0.14 [−0.3, 0.01]
9	You are alone at home and pass gas. It is silent but smells strongly.	0.67 [0.59, 0.75]	–	0.37 [0.2, 0.54]
-----------------------------------------------------------------------------------------------------------------------------				
2	You are standing next to a stranger and notice that the t-shirt they are wearing smells strongly from their sweat.	0.52 [0.46, 0.59]	0.64 [0.59, 0.68]	–
4	You are sitting next to a stranger and notice that their feet smell strongly.	0.51 [0.45, 0.57]	0.7 [0.66, 0.75]	–
6	You are chatting with a stranger and notice that their breath smells strongly.	0.51 [0.44, 0.57]	0.67 [0.63, 0.72]	–
12	You use the bathroom after a stranger and notice that the room smells strongly of their urine.	0.51 [0.45, 0.57]	0.55 [0.49, 0.61]	–
10	You are sitting next to a stranger and they pass gas. It is silent but smells strongly.	0.47 [0.41, 0.53]	0.62 [0.57, 0.67]	–
8	You use the bathroom after a stranger and notice that the room smells strongly of their feces.	0.46 [0.4, 0.52]	0.57 [0.51, 0.63]	–

Items are sorted form the highest to the lowest loading onto the general BODS. The dashed line marks the division between internal and external items. Std, standardized; CI, confidence interval.

#### 2.3.2 Measurement invariance for gender

The configural model showed a good fit (𝜒2 = 217.37, df = 72, CFI robust = 0.97, TLI robust = 0.95, RMSEA robust = 0.08, SRMR = 0.03). Even though the 𝜒^2^ difference test between the configural model and the metric model was statistically significant (Δ𝜒^2^ = 389.64, df = 21, *p* < 0.001), the other fit indices did not worsen substantially (ΔCFI robust = −0.005, ΔRMSEA robust = −0.004, ΔSRMR = 0.015). Even though the 𝜒^2^ difference test between the metric model and the scalar model was statistically significant (Δ𝜒^2^ = 407.51, df = 102, *p* = 0.044), the other fit indices did not worsen substantially (ΔCFI robust = −0.001, ΔRMSEA robust = −0.002, ΔSRMR = 0.002).

#### 2.3.3 Gender differences in the BODS

**Figure 2:**
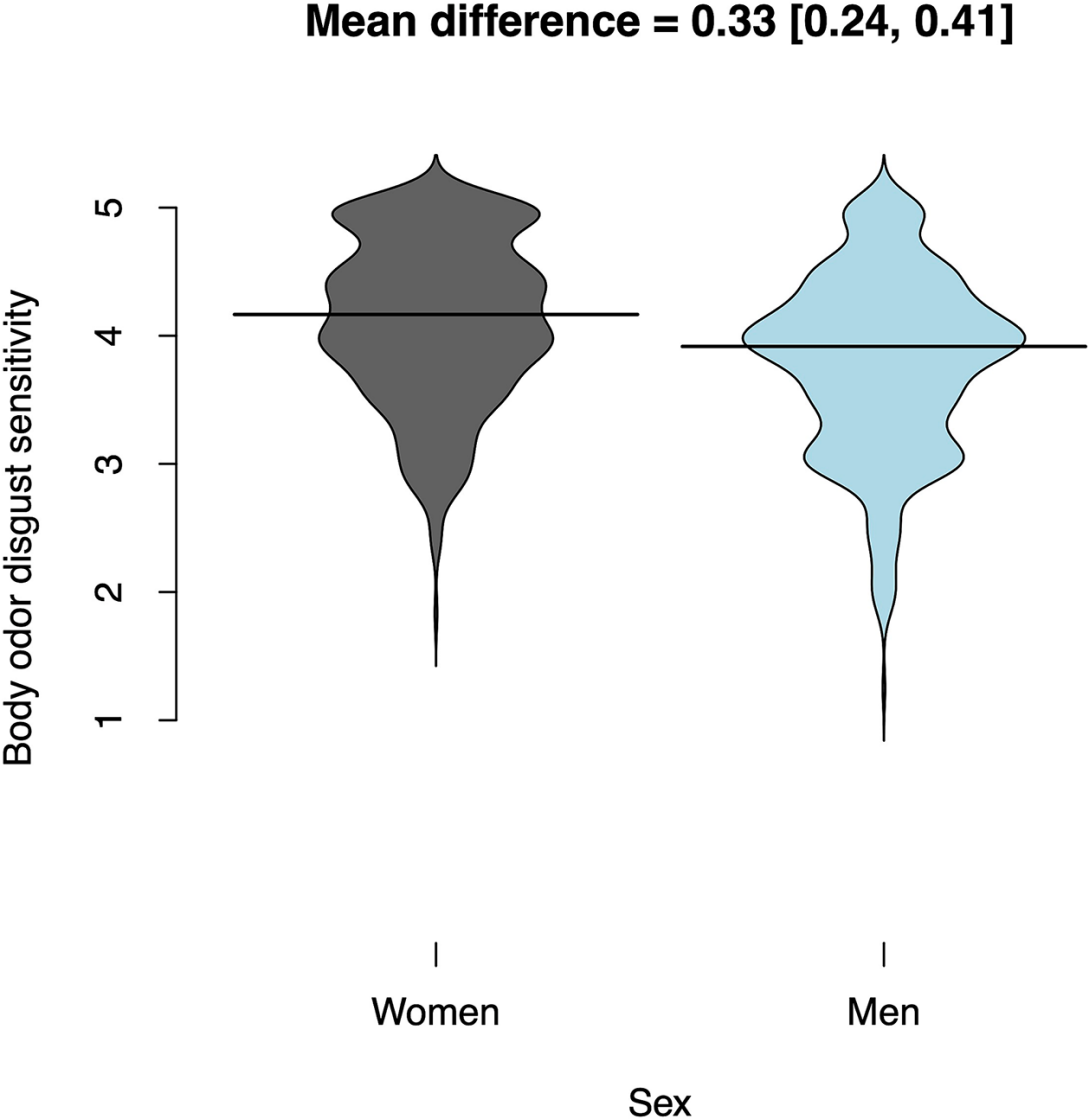
Sex differences in body odor disgust scale (BODS). The solid lines represent the medians.

Women rated their body odor disgust sensitivity on average 1/3 of a point higher than men (0.33 [0.24, 0.41]; *t*(895.71) = 7.37; *p* < 0.001; Cohen’s *d* = 0.49, Figure 2). Table 3 shows mean BODS scores for both sexes. The gender differences for both subscales were fairly similar: 0.28 [0.19, 0.37] on the internal scale and 0.38 [0.27, 0.48] on the external scale. The OSF folder^2^ includes plots for the gender differences on the two subscales.

**Table 3:** Mean (and standard deviation) BODS score by sex.

	**Female**	**Male**	**All**
BODS	4.11 (0.64)	3.78 (0.69)	3.94 (0.69)
Internal	3.83 (0.8)	3.45 (0.82)	3.64 (0.83)
External	4.38 (0.62)	4.11 (0.74)	4.24 (0.7)

These scores represent the (unweighted) means of all items within the scale or subscale.

### 2.4 Interim discussion

In our first study, we validated the BODS on a large and fairly representative sample from the Italian population. The CFA confirmed the structure found for the English version, and a multi-group CFA showed that the measure is configurally, metrically, and scalarly invariant to gender, so the means of the BODS could be confidently compared, showing slightly higher ratings for women compared to men.

## 3 Study 2

Based on the results of the first study, we aimed to cross-validate the Italian adaptation of the BODS from a dataset that is part of a larger project aimed at studying the relationship between disgust sensitivity, fear of COVID-19, social dominance orientation, and personality (more details, included the registration, can be found at the open Science Framework Page of the project^3^).

### 3.1 Materials and methods

#### 3.1.1 Participants

The data were collected between 03 May 2022 and 27 May 2022 through the Qualtrics platform, with the same specifications as in Study 1. All participants provided consent to participation. The median duration of the survey was 11 min and 46.5 s. Qualtrics recruited 402 participants who declared to reside in Italy and who completed the survey in Italian (*F* = 203).

#### 3.1.2 Measures

As in Study 1, we collected demographic information and the BODS. Furthermore, we collected the following measures to test for criterion and construct validity.

##### 3.1.2.1 Concern about the COVID-19 pandemic

To evaluate concern about the ongoing COVID-19 pandemic, we asked participants to rate how much they agree with the following four statements: (1) “It is important to clean my phone,” (2) “It is important to avoid being in close proximity to others outside my own household,” (3) “wearing face masks is important,” and (4) “coronavirus outbreak is a major threat for the health of the population of my country.” Answers were provided on a scale from 1 (*strongly disagree*) to 7 (*strongly agree*).

##### 3.1.2.2 Fearfulness

We used the fearfulness facet from the HEXACO personality inventory (Lee and Ashton, 2018) emotionality trait. Participants answered 4 questions on a scale from 1 (*strongly disagree*) to 7 (*strongly agree*).

##### 3.1.2.3 The three-domain disgust scale: pathogen subscale

We used the pathogen subscale from the three-domain disgust scale (TDDS, Tybur et al., 2009) validated for the Italian population (Poli et al., 2019). Participants answered 6 questions related to how disgusting they feel about scenarios that involve a potential pathogen threat (e.g., seeing mold on food). Answers were provided on a scale from 1 (*Not at all disgusting*) to 7 (*Extremely disgusting*).

##### 3.1.2.4 Attention check

We included a similar attention check as in Study 1, this time instructing participants to type ‘time’ in the same text box, after reading the same paragraph about free-time activities. In total, 34 failed the attention check and were therefore removed from the analyses.

#### 3.1.3 Data analysis

##### 3.1.3.1 Confirmatory factor analysis

We followed the same procedure described in Study 1.

##### 3.1.3.2 Construct and criterion validity

We tested the convergent validity of BODS by looking at its relationship with the score on the TDDS pathogen subscale, and to test the discriminant validity, we looked at its relationship with fearfulness. To estimate the relationships, we used Bayesian parameter estimation and modeling, as implemented in the *rethinking* package (McElreath, 2023). We first standardized the variables and then modeled the ratings using regularizing priors and normal distribution (*M* = 0, SD = 0.5) for the intercept and beta coefficients, and an exponential distribution for the sigma parameter. We provide coefficient estimates with 94% (see McElreath, 2018, p. 56 for recommendations against using 95%) highest posterior density interval (HPDI). Additionally, we plot the posterior estimates for the two relationships to visualize a potential overlap or lack thereof. No or small overlap would suggest that the two relationships (between BODS and TDDS, and between BODS and Fearfulness) are different from each other.

Using the same approach, we further tested the criterion validity of BODS by investigating its relationship with concern about the COVID-19 pandemic. We also investigated the relationship between COVID-19 concerns and TDDS. Concerns about COVID-19 should be positively associated with BODS (as well as the TDDS) if these constructs all rely on one pathogenic avoidance mechanism such as outlined in the BIS framework. Additionally, we included both TDDS and BODS in one model to see if BODS shows incremental predictivity over TDDS. Here we referred to information criteria [widely applicable information criterion (also known as the Watanabe–Akaike information criterion)], WAIC to compare models including TDDS pathogen, BODS, or both. For a model to be considered better, it had to have a lower WAIC value than the alternative model(s), and this difference (ΔWAIC) had to be at least twice as big as the standard error of the difference (ΔSE). Note that to model the effect of these two variables on COVID-19 concerns, we also included information about participants’ age, gender, and education.

### 3.2 Results

In the final sample (*N* = 368, *F* = 186) the age spanned from 18 to 69 (mean = 44.53, SD = 13.36). The age distribution somewhat reflected the distribution of the Italian population (see Table 1), as retrieved from ISAT 2023^4^ (see Table 1), even though the group aged 55+ was slightly under-represented. In terms of educational attainment, the sample has a remarkable proportion of people with at least a bachelor’s degree (35.8%). Narrowing the analysis only to the participants aged 18–64, we found that the percentage of the sample holding a degree is higher (36.1%) than the proportion in the Italian population aged 16–64 years (20.1%, source: ISTAT). Table 4 shows descriptives for the variables of interest for men and women.

**Table 4:** Descriptive statistics (mean and standard deviations in parentheses) for the body odor disgust sensitivity scale (BODS, the total score as well as the scores on the two subscales), Three domain disgust scale – pathogen subscale (TDDS pat), the fearfulness facet of the emotionality domain of the HEXACO, and concern about COVID-19 pandemics scale (COVID-19 concern).

	**Female**	**Male**	**All**
BODS	3.6 (0.73)	3.75 (0.76)	3.68 (0.74)
BODS internal	3.54 (0.75)	3.69 (0.78)	3.62 (0.77)
BODS external	3.66 (0.75)	3.81 (0.78)	3.73 (0.77)
TDDS pat	4.81 (1.08)	4.98 (1.06)	4.89 (1.07)
Fearfulness	3.66 (0.94)	3.81 (1.11)	3.73 (1.05)
COVID-19 concern	3.66 (1.52)	3.81 (1.49)	3.73 (1.51)

These scores represent the (unweighted) means of all items within the scale or subscale.

#### 3.2.1 CFA on BODS

As in Study 1, the Mardia test for multivariate normality showed that our data were not normally distributed both in terms of skewness (942.76, *p* < 0.001) and kurtosis (21.3, *p* < 0.001). Therefore, we used a maximum likelihood robust (MLR) estimator. We encountered convergence issues when applying the same model as in Study 1. These issues were related to one of the items, which was extremely skewed, with a median of 5 (maximum on the scale) and 75% of the answers above 4. To solve the convergence issues in this smaller sample size, we removed this item, as well as its corresponding item on the internal subscale (see Table 5 for included items). The updated CFA model showed a good fit (𝜒2 = 26.16, df = 20, CFI robust = 1, TLI robust = 0.99, RMSEA robust = 0.03, SRMR = 0.01). On top of the good global fit, the local fit looked acceptable as well, as all the items had loadings on the general factor ranging from −1 to 1. In terms of reliability, the scale showed excellent internal consistency (𝜔*_t_* = = 0.93, 𝜔_ℎ_ = 0.73). Using recommendations from Reise et al. (2013), we find support for the existence of a general factor (𝜔_ℎ_ =  = 0.74, above 0.70) and ECV (ECV = 0.65, above 0.60). Similar to Study 1, the PUC value is lower than the threshold suggested (PUC = 0.56, below 0.90).

**Table 5:** Item loadings onto the general factors (BODS, body odor disgust sensitivity) and the specific factors: external and internal BODS in Study 2.

**#**	**Item**	**Estimate (std)** **95% CIs**		
		BODS	External	Internal
2	You are standing next to a stranger and notice that the t-shirt they are wearing smells strongly from their sweat.	0.73 [0.31, 1.15]	0.42 [−0.25, 1.08]	–
11	While alone at home, you use the bathroom. Afterwards, you notice that the room smells strongly of your urine.	0.71 [0.21, 1.21]	–	0.36 [−0.5, 1.22]
3	You are alone at home and notice that your feet smell strongly.	0.69 [0.21, 1.18]	–	0.47 [−0.25, 1.19]
4	You are sitting next to a stranger and notice that their feet smell strongly.	0.68 [0.26, 1.1]	0.48 [−0.12, 1.07]	–
5	You are alone at home and notice that your breath smells strongly.	0.67 [0.4, 0.95]	–	0.39 [−0.1, 0.87]
1	You are alone at home and notice that the t-shirt you are wearing smells strongly from your own sweat	0.64 [0.33, 0.96]	–	0.54 [0.24, 0.84]
12	You use the bathroom after a stranger and notice that the room smells strongly of their urine.	0.62 [0.25, 0.99]	0.48 [0.01, 0.95]	–
6	You are chatting with a stranger and notice that their breath smells strongly.	0.59 [0.15, 1.03]	0.5 [0, 0.99]	–
10	You are sitting next to a stranger and they pass gas. It is silent but smells strongly.	0.59 [0.17, 1]	0.56 [0.15, 0.98]	–
9	You are alone at home and pass gas. It is silent but smells strongly.	0.56 [0.12, 1]	–	0.52 [0.03, 1.02]
	Removed items			
8	You use the bathroom after a stranger and notice that the room smells strongly of their feces.			
7	While alone at home, you use the bathroom. Afterwards, you notice that the room smells strongly of your feces.			

Items are shown in order based on highest to lowest loading for general BODS. std, standardized; CI, confidence interval.

#### 3.2.2 Convergent and discriminant validity: the BODS is positively related to the TDDS pathogen subscale, but not related to fearfulness

**Figure 3:**
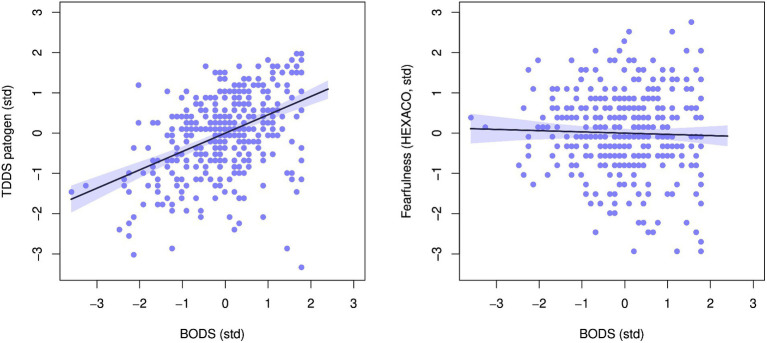
Relationship between body odor disgust scale (BODS) and the pathogen subscale of the Three domain disgust sensitivity scale (TDDS, left) and fearfulness facet of the HEXACO personality inventory (right). The plots show standardized values. The line represents the mean effect with 94% HDPI (shaded area).

**Figure 4:**
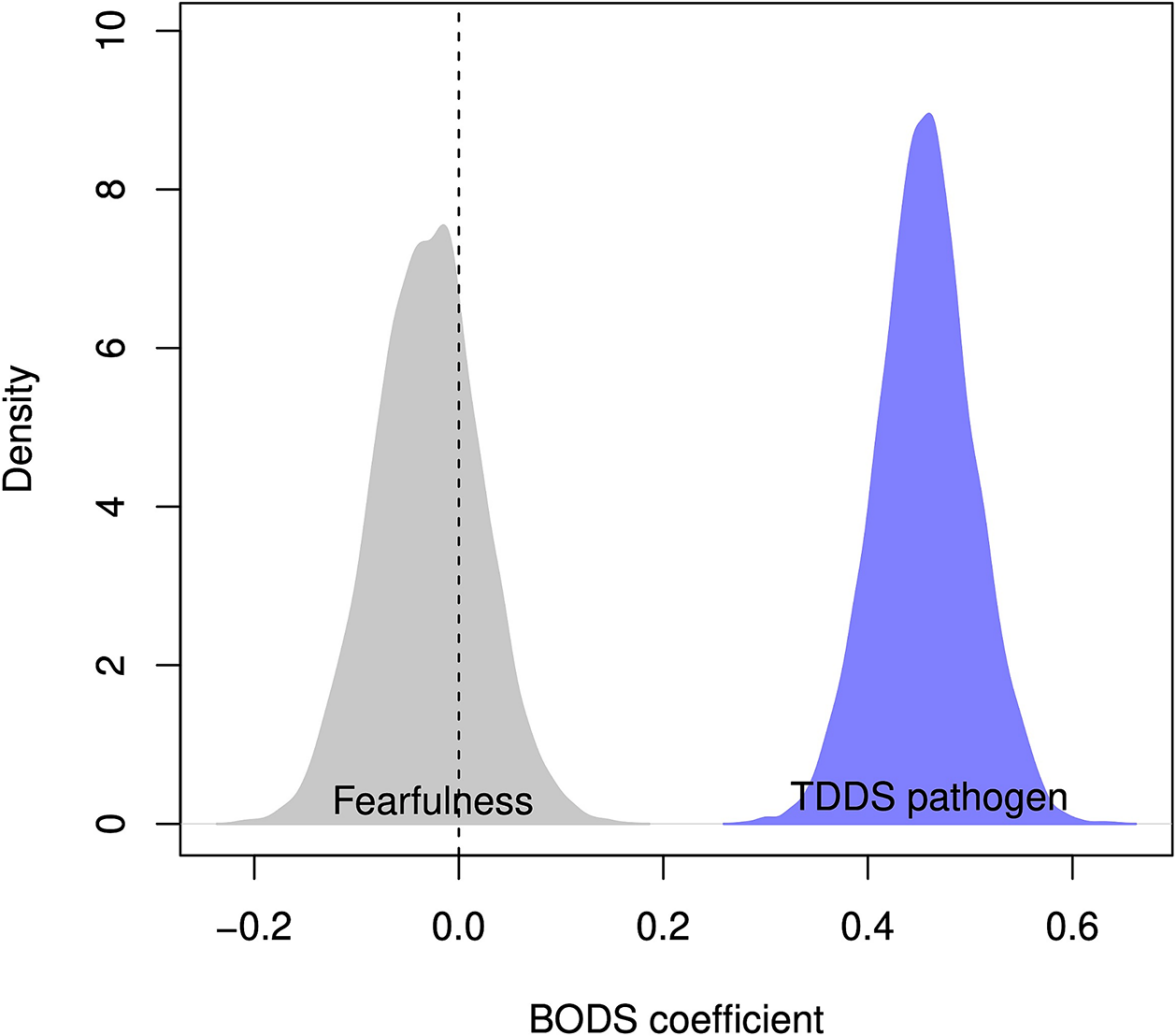
Posterior distribution of estimated coefficient of relationship between BODS fearfulness (gray) and TDDS pathogen (blue). The dashed vertical line represents no relationship.

The BODS was positively related to the TDDS pathogen score (0.46 [0.38, 0.53 94% Highest Density Prediction Intervals (HDPI)], Figure 3, left panel), confirming the convergent validity assumptions. In contrast, the BODS was not related to the fearfulness score (−0.03 [−0.12, 0.05 94% HPDI], Figure 3, right panel), providing support for discriminatory validity. The relationship between the BODS and TDDS was stronger than that with fearfulness, and the posterior distributions of the two effects did not overlap (Figure 4).

#### 3.2.3 Both BODS and TDDS are positively related to concern about COVID-19

**Figure 5:**
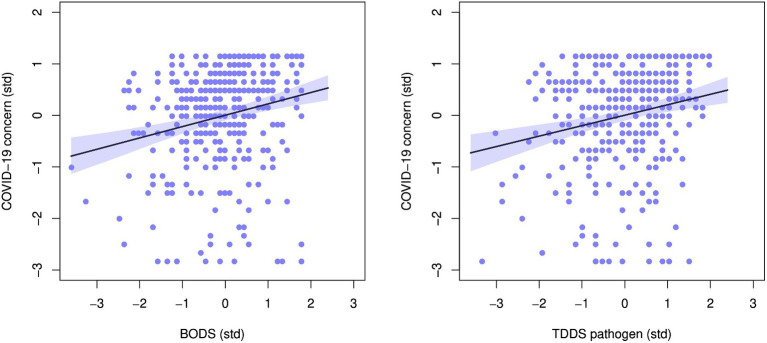
Relationship between concern about COVID-19 and: **(A)** Body odor disgust scale (BODS, left), **(B)** the pathogen subscale of the three domains disgust sensitivity scale (TDDS, right). The plots show standardized values. The line represents the fit line with 94% HDPI (shaded area).

**Figure 6:**
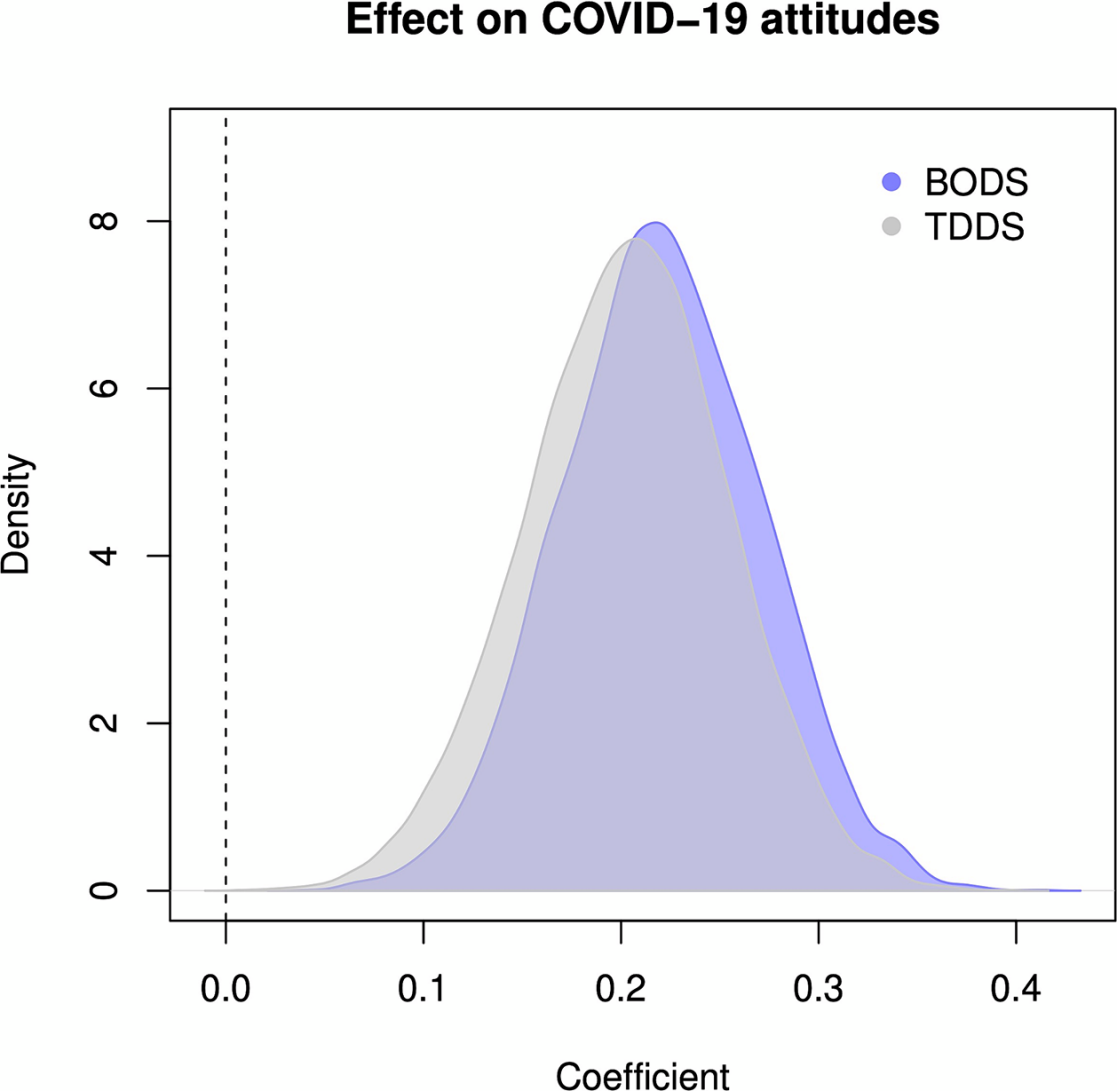
Posterior distribution of the estimate coefficient of the relationship between COVID-19 concern and **(A)** BODS (blue), **(B)** TDDS pathogen (gray). The dashed vertical line represents no relationship.

The BODS was positively related to concern about the COVID-19 pandemic (0.22 [0.14, 0.30 94% HDPI], Figure 5, left panel), and so was the TDDS pathogen (0.20 [0.12 0.28] 94% HDPI). Both were related to COVID-19 concerns similarly, as illustrated by the highly overlapping posterior distribution of the coefficient estimates (Figure 6). When included in one model, the estimates for the effects of BODS and TDDS pathogen were both slightly smaller (BODS = 0.16 [0.07, 0.25 94% HDPI]; TDDS pathogen = 0.13 [0.04, 0.22 94% HDPI]). All three models performed similarly regarding information criteria: ΔWAICs <= 5.7, with ΔSEs > = 5.51. Thus, we did not find support for the incremental predictivity of BODS.

## 4 Discussion

In the present study, we adapted and validated in Italian the body odors disgust scale (BODS, Liuzza et al., 2017a,b) in two large samples that were relatively representative of the Italian population.

In our first study, a confirmatory factor analysis (CFA) validated the hypothesized bifactor structure l. Although this choice is different from the one pursued in the first validation paper, it is conceptually similar, since it recognizes at the same time the existence of a general factor and the dimensionality underlined by the source dimension. The bifactor model has the advantage of partitioning the variance due to the general factor and the variance due to specific factors, which can be seen as manifestations of the constructs. In our case, we found that the general factor, overall, is strongly related to the observed items, even though it manifests through specific factors related to internal and external sources of the odors, with the latter being more specific, since it explains a larger part of the variance not explained by the general factor among the related items (Reise et al., 2010).

To compare groups on their average scores, it is crucial to prove that the measurement works in the same way among these groups, namely to establish measurement invariance. In particular, in order to compare averages in observed scores, it is crucial to attain at least scalar invariance. This means that the instrument must not only exhibit the same factor structure (configural invariance) and factor loadings (metric invariance) across groups, but also invariant intercepts. Without this, differences in observed scores could be artefactual (Meredith and Teresi, 2006). Conversely, a lack of difference may mask a difference in the latent means. Investigating measurement invariance is thus critical, yet often overlooked (Flake et al., 2017; Hussey and Hughes, 2020; Maassen et al., 2023). In our study, we pursued and conducted a multiple-group confirmatory factor analysis (MGCFA) and found that the Italian version of the BODS is metrically invariant across genders, allowing us to compare differences between men and women in their BODS scores. Our findings showed that women displayed higher levels of pathogen disgust than men. Although this gender difference is of a smaller magnitude compared to the marked difference that can be found in sexual disgust (see Al-Shawaf et al., 2018), such a difference seems to be constantly found in the literature on the BODS (Liuzza et al., 2017a,b) and other measures measuring pathogen disgust (Olatunji et al., 2007; Tybur et al., 2009, 2016), including in Italian samples (Mancini et al., 2001). Although it is hard to prove that this gender difference has evolutionary roots, several findings point in that direction. For instance, in a recent reanalysis of the data coming from a large-scale data collection involving 31 different countries (Tybur et al., 2016), we found that not only is such a gender difference generalizable across countries, but it does not seem to covary with reliable indices of gender equality (Liuzza and Occhiuto, 2024). Furthermore, data coming from primates show a sex difference in disease avoidance behaviors: females of Japanese macaques, compared to males, spend more time washing potatoes that had been previously contaminated by feces (Sarabian and Mac Intosh, 2015). However, the distal explanation for such a difference has not yet been clarified, and many evolutionary hypotheses may account for this difference. Some of the most compelling evolutionary hypotheses rest on the fact that mothers’ health matters more, as they also provide for the survival of their kinship (Al-Shawaf et al., 2018). This hypothesis resonates with evidence that disgust sensitivity increases during pregnancy (Dlouhá et al., 2024), and that pregnant women displayed even higher levels of disgust during the COVID-19 pandemic.

The second study cross-validated the results from Study 1, even though we had to remove two items for convergence issues, as one of them had a marked ceiling effect which, paired with the smaller sample size, hampered the estimation. On top of the satisfactory results of the global fit, an inspection of the local fit of the bifactor model revealed that all the items loaded adequately on the general factor, and this observation was corroborated by the satisfactory level of the hierarchical omega reliability index, which assesses how strongly the items are related to the general factor. This time, the loadings onto the specific factors (internal vs. external source) varied to a greater extent. However, it should be kept in mind that the latter values assess how much these items load into the specific factors after accounting for the variance explained by the general factor.

In the second study, we also provided evidence that the BODS has convergent validity with other well-established measures of pathogen disgust sensitivity such as the pathogen subscale of the TDDS (Tybur et al., 2009), but also discriminant validity with the fearfulness facet of the emotionality domain of the HEXACO. This result confirms that even though disgust sensitivity is somewhat related to a general proneness to experience negative emotions, it taps into a distinguishable construct (Tybur et al., 2018).

In the second study, we also provided some evidence for the criterion validity by showing that the BODS would predict disease avoidance behavior as measured by measuring the attitudes toward behaviors aimed at minimizing the risk of COVID-19 contamination. However, the BODS did not show incremental validity as compared to the pathogen subscale of the TDDS, another widely used measure of disgust. This result is partially at odds with what we found in a previous BODS validation study (Liuzza et al., 2017a). However, in that study, we used a different alternative measure of pathogen disgust (the DS-R, Olatunji et al., 2007), and a different measure of BIS, the perceived vulnerability to disease (PVD, Duncan et al., 2009), which represents another trait measure rather than a measure of behavioral intentions and attitudes toward a specific pathogen threat. Moreover, it is not obvious how and which subsets of the behavioral immune system should have been activated by the COVID pandemic (Ackerman et al., 2021), which occurred in an environment that is quite different from the one in which the BIS has evolved. For instance, we now live in crowded environments, and pathogens such as SARS-COV2 are airborne and can spread from asymptomatic infected individuals. In this context, the part of the BIS activated by body odors may not have played any role in a pandemic response other than a general pathogen avoidance behavior that the BODS shares with other measures. In particular, the main vectors of infection in COVID (invisible droplets from asymptomatic individuals) hardly produce body odor elicited disgust.

A possible limitation of the current study is that, even though the two samples were representative of the population in terms of gender and, to a lesser extent, age, they were not representative in terms of education. However, in this study, we did not aim to provide cut-offs and norms for using this instrument in assessment settings, and therefore we are confident that this limitation does not threaten the validity of our conclusions.

Another possible limitation is that, even though participants were recruited through Qualtrics, we cannot rule out whether some of them might have used AI to respond to the survey, although this is unlikely, since we used an attention check that helped us screen out unreliable, and even potentially AI-generated, responses (Webb and Tangney, 2022). Finally, even though we aimed at a nationally representative sample, in study one the elderly population was over-represented. However, different segments of the population were still fairly represented given the sample size.

Taken together, these results that the Italian adaptation of the BODS can be used in the Italian population with good validity and reliability.

## Data availability statement

Publicly available datasets were analyzed in this study. This data can be found here: https://osf.io/qr7vn/.

## Ethics statement

The studies involving humans were approved by Etikprövningsmyndigheten (2018/1169-31/5 and 2020-04690). The studies were conducted in accordance with the local legislation and institutional requirements. The participants provided their written informed consent to participate in this study.

## Author contributions

ML: Conceptualization, Formal analysis, Funding acquisition, Methodology, Writing – original draft, Writing – review & editing. MZ: Data curation, Formal Analysis, Investigation, Writing – original draft, Writing – review & editing, Methodology. JO: Conceptualization, Writing – review & editing, Methodology.

## Footnotes

^1^http://dati.istat.it/

^2^https://osf.io/qr7vn/

^3^https://osf.io/ac6dh

^4^http://dati.istat.it/
